# A Case of Hypophosphatasia Started Enzyme Replacement Therapy Since Babyhood Stage

**DOI:** 10.3390/children12010061

**Published:** 2025-01-06

**Authors:** Tatsuya Akitomo, Noriko Niizato, Ami Kaneki, Masashi Ogawa, Taku Nishimura, Mariko Kametani, Momoko Usuda, Yuko Iwamoto, Chieko Mitsuhata, Ryota Nomura

**Affiliations:** 1 Department of Pediatric Dentistry, Graduate School of Biomedical and Health Sciences, Hiroshima University, 1-2-3 Kasumi, Minami-ku, Hiroshima 734-8553, Japan; kaneki@hiroshima-u.ac.jp (A.K.); caries0@hiroshima-u.ac.jp (M.O.); nishi04@hiroshima-u.ac.jp (T.N.); mrysk25@hiroshima-u.ac.jp (M.K.); musuda@hiroshima-u.ac.jp (M.U.); yuko-tulip@hiroshima-u.ac.jp (Y.I.); chiekom@hiroshima-u.ac.jp (C.M.); rnomura@hiroshima-u.ac.jp (R.N.); 2 Acacia Kids’ Dental Clinic, Hiroshima 731-0102, Japan; acacia.pedo.dc@gmail.com

**Keywords:** hypophosphatasia, enzyme replacement therapy, tooth exfoliation

## Abstract

Background: Hypophosphatasia (HPP) is an inherited disease caused by low activity of tissue-nonspecific alkaline phosphatase. Dental characteristics include premature loss of primary teeth, enlarged pulp chambers, and enamel hypoplasia. Although enzyme replacement therapy with asfotase alfa was approved in 2015, there are few reports about the dental outcomes of this treatment. Case presentation: A 1-year-old girl referred to our hospital had already lost two primary teeth at the time of her initial visit. She started enzyme replacement therapy 6 days after birth, and genetic analysis later confirmed the diagnosis of HPP. At the age of 4 years and 7 months, 11 primary teeth had been lost, and some of the exfoliated teeth showed inflammatory root resorption or root fracture. There was also a history of abscess formation in a non-carious primary molar. Conclusions: This report suggests that early enzyme replacement therapy may prevent traditional tooth loss in patients with HPP. It also highlights the new challenges posed for dental professionals in providing infection control in large pulp cavities and receding periodontal tissue.

## 1. Introduction

Hypophosphatasia (HPP) is a genetic disorder resulting from a deficiency in tissue-nonspecific alkaline phosphatase (TNSALP), an enzyme essential for proper mineralization. This metabolic disorder was first identified by John C in 1948 [1]. The reduced activity of TNSALP leads to the accumulation of its substrates, including inorganic pyrophosphate, a potent inhibitor of mineralization, and pyridoxal-5′-phosphate, a crucial cofactor for various enzymes. This accumulation disrupts normal musculoskeletal and systemic function, causing the characteristic symptoms of HPP [2].

The clinical presentation of HPP is highly variable, ranging from the severe, often fatal perinatal form to milder, adult-onset forms, which may present with nonspecific symptoms like joint problems and musculoskeletal pain [3]. The condition is typically classified by the age of onset and the presence or absence of bone-related symptoms, with categories including perinatal, prenatal benign, infantile, childhood, adult, and odontohypophosphatasia [3]. Mutations in the ALPL gene, responsible for producing TNSALP, lead to the enzyme’s reduced activity, with over 400 known mutations (mostly missense) inherited through either autosomal dominant or recessive patterns [2]. The incidence of severe forms of the disease is rare, with reported rates ranging from 1 in 100,000 live births in Canada to 1 in 300,000 in Europe. In Japan, the incidence of perinatal severe HPP is 1 in 368,640 [2,4].

Oral and dental features of HPP patients include the early and spontaneous loss of the lacteal teeth before the age of 3 years and possible loss of permanent teeth. Therefore, dental manifestations, such as early exfoliation of primary teeth, sometimes lead to a diagnosis of mild HPP [5,6]. It is associated with infantile, childhood, and odontohypophosphatasia due to disturbed cementum formation [7,8]. Foster et al. (2012) reported that excess inorganic pyrophosphate in alkaline phosphatase knockout mice disturbed cementum formation and caused root avulsion, which is consistent with premature tooth loss in the human condition hypophosphatasia, and there are many studies being conducted to clarify this mechanism [9,10,11]. The loss of teeth can cause esthetic–functional psychological problems and dysarthria of speech sounds [12,13]. Therefore, prosthodontic treatment with a partial denture is recommended for patients with exfoliated teeth [6,14]. In addition, mouse studies demonstrated that ameloblasts, odontoblasts, cementoblasts, osteoblasts, and periodontal ligament cells express TNSALP [15]. HPP patients have other common characteristics, including enlarged pulp chambers, enamel hypoplasia, and tooth eruption disorders, such as late tooth eruption and ankylosis of primary teeth [16].

Rickets is a disorder characterized by a generalized impairment of mineral deposition into the matrix of the growing skeleton [17,18]. Clinical symptoms of rickets are bone deformity, spinal curvature, craniotabes, enlargement of the anterior fontanel, rachitic rosary, and joint swelling [19]. In HPP, extracellular accumulation of inorganic pyrophosphate often leads to rickets [18]. X-linked hypophosphatemia (XLH) is the most common genetic cause of rickets, and the radiographic examination shows large pulp chambers, devitalized teeth, and generalized alveolysis [20,21]. It is reported that an XLH patient can have recurrent alveolar abscesses with fistulas; on the other hand, there are few reports regarding its occurrence in the HPP patient [22].

The TNSALP protein (asfotase alfa) was first described in 2008, and subsequent success in treating patients with severe forms of the disease was reported in 2012 [23]. In July 2015, asfotase alfa was approved in Japan, followed by the EU and Canada in August that year and by the US Food and Drug Administration in October [23,24]. In life-threatening perinatal and infantile HPP, asfotase alfa therapy significantly enhances the survival rate at years 1 and 5, and it has been proven to be a significant and sustainable treatment for patients of any age group suffering from HPP [25,26].

In the oral cavity, it has been reported that the process of premature loss of primary teeth is stabilized in children receiving enzyme replacement therapy with asfotase alfa [16]. Schroth et al. (2021) reviewed the development and exfoliation patterns of primary and permanent teeth and suggested that asfotase alfa may limit the mobility of primary teeth in HPP patients, although it is important to note the wide age range and limited evidence reported [27]. Given the scarcity of reports about the dental outcomes of asfotase alfa treatment in HPP, it is clear that further research is required.

We encountered a patient who was diagnosed with HPP and had started asfotase alfa therapy 6 days after birth. This report describes the patient’s dental condition, including exfoliation and oral management.

## 2. Detailed Case Description

A girl aged 1 year and 8 months was referred to our hospital for oral management by the dental department of the general hospital where she had regular checkups for HPP. She was born at 38 weeks gestation with a height of 48.5 cm and a weight of 2608 g. The patient was wheezing, and a decrease in SpO2 was detected 2 h after birth, resulting in admission to the neonatal intensive care unit. Although no abnormalities were found by visual examination, X-ray examination revealed hypocalcification of bones throughout the body, and blood tests showed an ALP level of 10 U/L, leading to a possible diagnosis of HPP. The patient was given vitamin B6, 10 mg/kg/day, for the prevention of epilepsy at 4 days after birth. At 6 days after birth, enzyme replacement therapy with asfotase alfa, 2 mg/kg three times a week, was started. Genetic analysis later confirmed the diagnosis of HPP. At her first visit, the patient’s respiratory condition was stable, and she was not using a ventilator.

**Figure 1:**
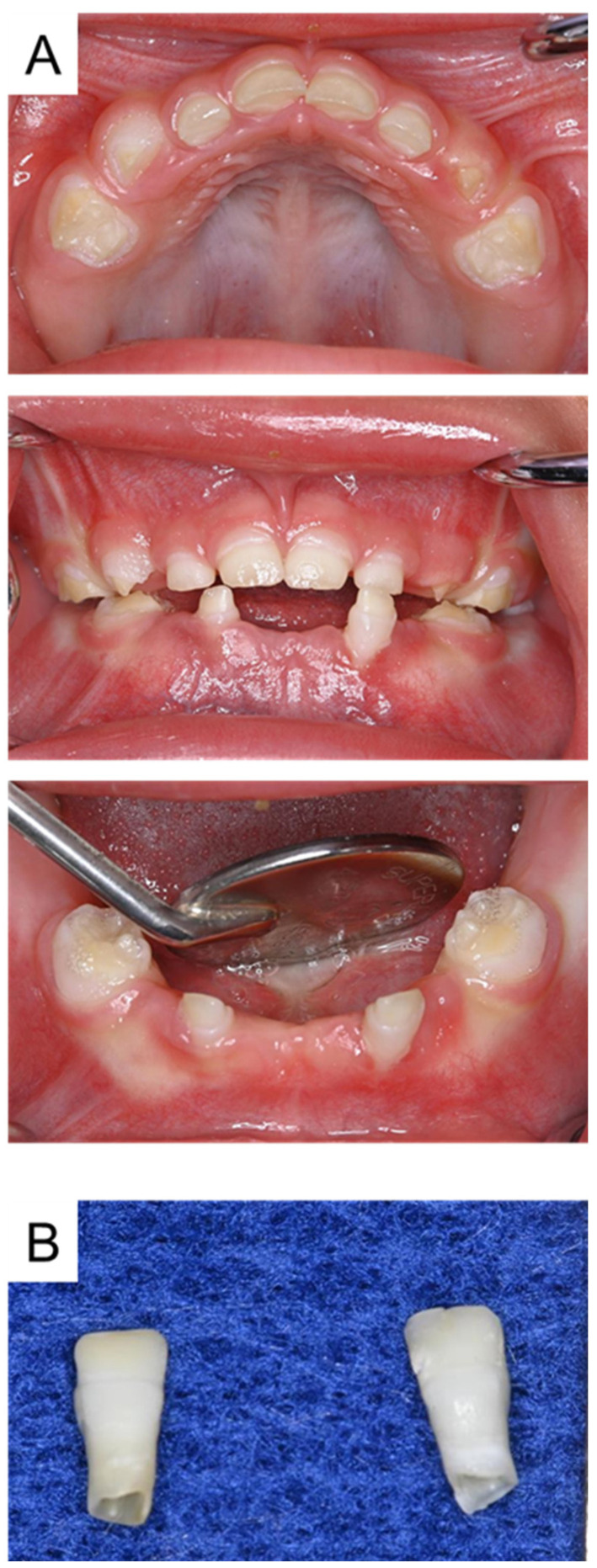
Intraoral photographs at the age of 1 year and 10 months (**A**) and an image of the exfoliated mandibular primary central incisors (**B**).

Intraoral examination showed 12 primary teeth in the oral cavity, and enamel hypoplasia was detected on a fully erupted tooth. The mandibular primary canines had not erupted. Intraoral photographs at 1 year and 10 months are shown in Figure 1A. Additionally, the mandibular left primary central incisor had fallen out at the age of 1 year and 3 months, and the right one had fallen out at 1 year and 7 months (Figure 1B). There was no pathological finding; therefore, we continued to monitor the patient regularly. Although the hypoplasia was not symptomatic, it was unesthetic and difficult to clean, so we restored it with a glass-ionomer liner (Vitrebond, 3 M Dental products, St Paul, MN, USA) during a follow-up appointment [28].

**Figure 2:**
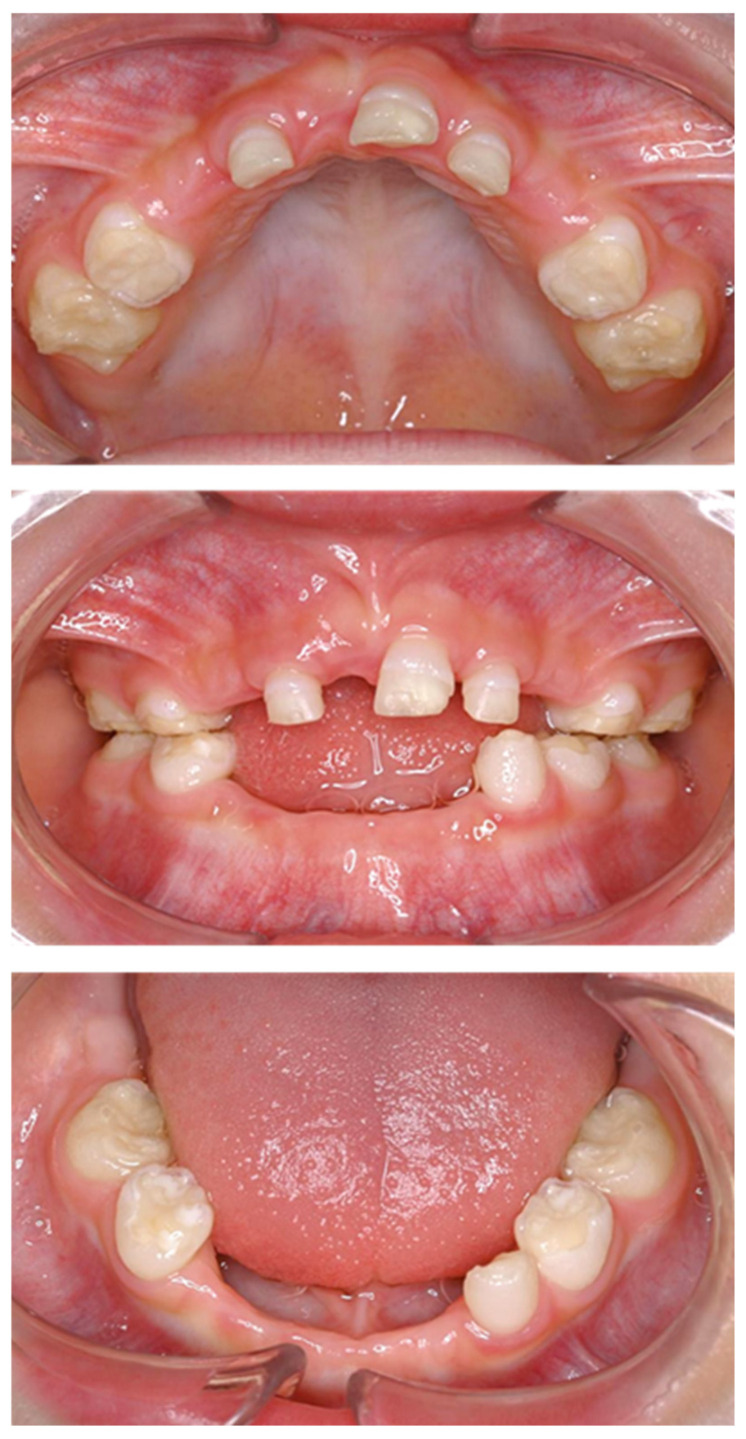
Intraoral photographs at the age of 3 years and 4 months.

**Figure 3:**
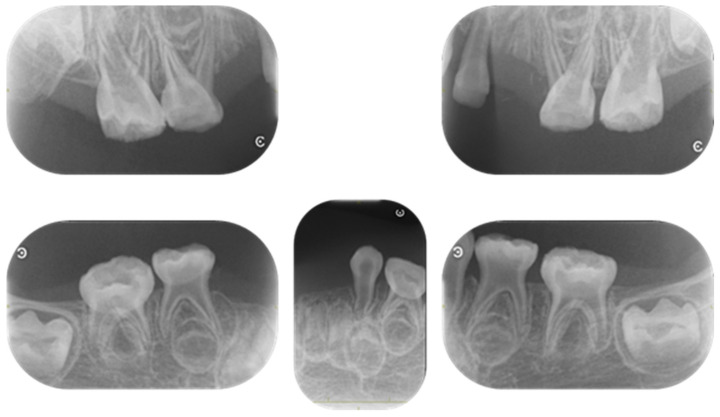
Periapical radiographs at the age of 3 years and 4 months.

Four primary teeth were lost at 2 years, and two new primary teeth were lost by the age of 3 years and 4 months (Figure 2). Additionally, radiographic examination revealed the enlarged pulp chambers characteristic of HPP (Figure 3).

**Figure 4:**
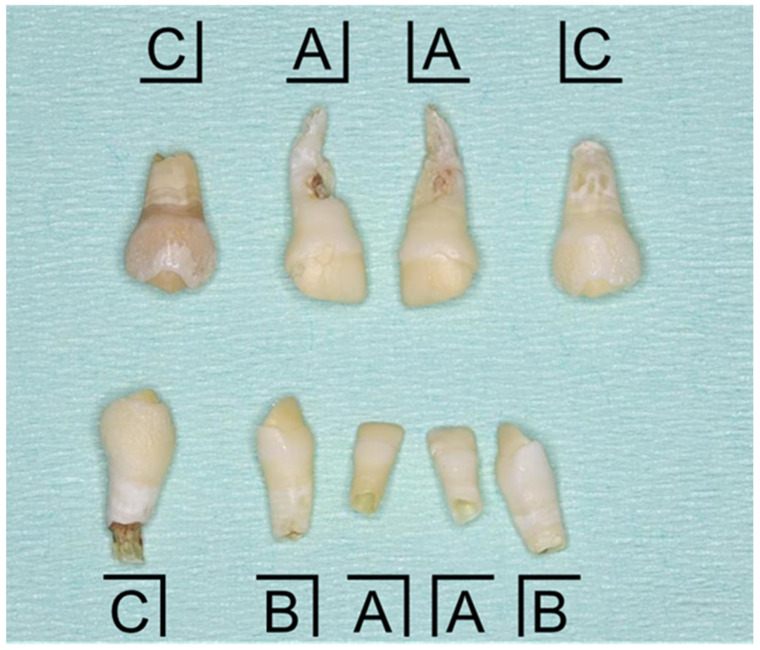
Intraoral photograph of exfoliated teeth at the age of 3 years and 10 months.

The maxillary left primary central incisor was lost at 3 years and 9 months, and nine primary teeth had already been exfoliated at that point (Figure 4). A partial denture was inserted 2 months later.

**Figure 5:**
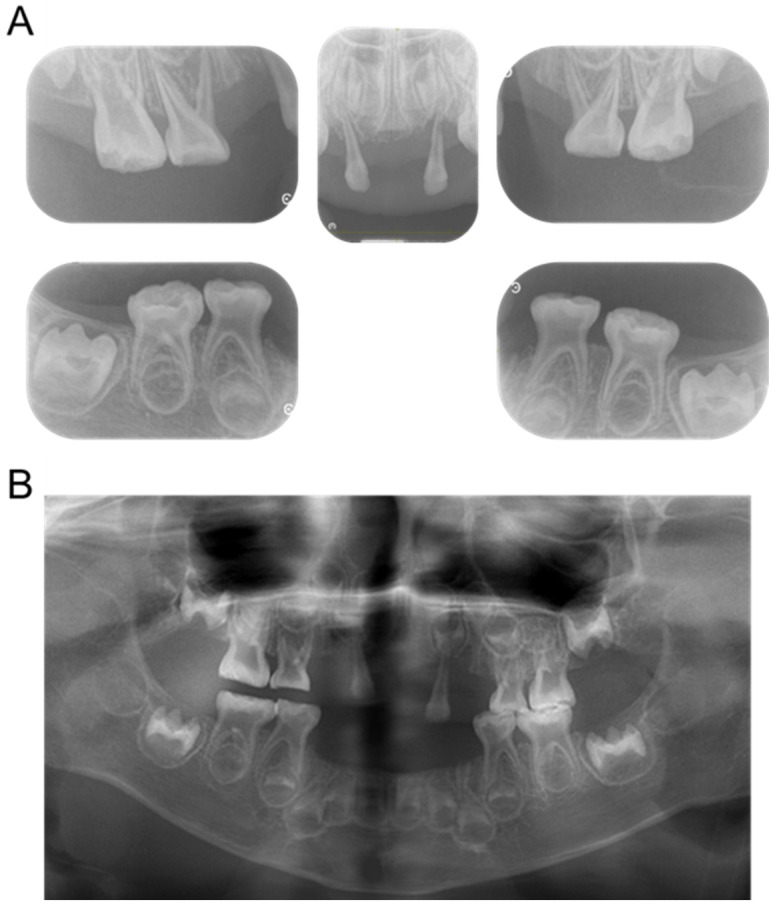
Radiographs taken at the age of 4 years and 4 months: periapical radiographs (**A**) and panoramic radiographs (**B**).

**Figure 6:**
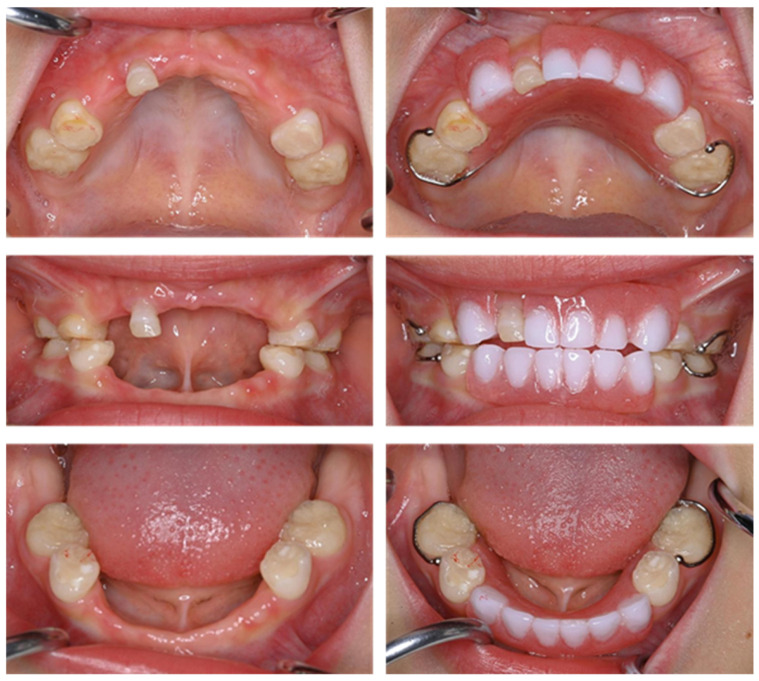
Intraoral photographs at the age of 4 years and 7 months.

At the age of 4 years and 4 months, the patient felt spontaneous pain, and an alveolar abscess was detected in the maxillary right primary first molar region; however, there were no carious teeth. A radiographic examination was taken, and the panoramic instrument was SOLIO XZ Ⅱ (ASAHIROENTGEN IND. Co., Ltd., Kyoto, Japan), and the scanning protocol involved a voltage of 64 kV and a current of 12 mA. It showed resorption of the alveolar bone around the primary first molar (Figure 5). External root resorption and alveolar bone resorption were also observed in the maxillary left primary lateral incisor. No changes were observed in the pulp cavity of the primary molars compared with 1 year earlier, and the roots of both the maxillary and the left mandibular primary canines that were thought to have been exfoliated were confirmed. We performed root canal treatment of the maxillary left primary first molar and incision of an abscess under local anesthesia. After the dental procedure, antibiotics and analgesics were prescribed, and the pain resolved at the time of the next visit. Considering the effect on the opposing tooth, the tooth was then restored with glass-ionomer cement (FUJI II LC, GC Corporation, Tokyo, Japan). As the maxillary left primary lateral incisor had a poor prognosis and the patient wished to allow it to fall out naturally, we continued to monitor the tooth until it was exfoliated 2 months later. Although 11 primary teeth had been lost by the age of 4 years and 7 months, no dysfunction was observed thanks to the use of the partial dentures (Figure 6). We are continuing with long-term follow-up of the patient.

## 3. Discussion

Dental caries and periodontitis are the most common oral diseases caused by oral microbiota and are major causes of tooth loss [29,30,31]. In addition to these diseases, pediatric dental professionals encounter a variety of patients with dental abnormalities, dental trauma, and malocclusion [32,33,34,35,36,37]. Dental abnormalities may or may not be associated with systemic diseases [19,38,39,40]. A typical dental finding of HPP is early exfoliation of primary teeth, and the diagnosis of HPP is often made on the basis of this manifestation [6,41]. In cases of HPP where teeth have been lost, long-term oral management, including the provision of dentures, is important [6,14].

In the present case, the patient was already diagnosed with HPP. Enamel hypoplasia and enlarged pulp chambers were detected, both of which are characteristic of HPP [16,42]. Oral photographs of the exfoliated teeth are shown in Figure 4, and the timeline is shown in Table 1. We compared the root completion time of our patient with the mean root completion time of children [43]. The mean root completion time of the primary central incisor is 1 year and 5 months, and the patient’s mandibular central incisor exfoliated at 1 year and 3 to 7 months; however, no root was found on the exfoliated tooth. The roots of the maxillary primary central incisors that were lost at 3 years of age were identified; however, external resorption was observed on the root surface. The mean root completion time of primary lateral incisors is from 1 year and 5 months to 2 years, and, as with the mandibular central incisor, no root was found on the mandibular right primary lateral incisor that was lost at the age of 2 years and 4 months. Radiographic examination of the mandibular left primary canine, which was exfoliated 1 year after the root completion time, revealed that the root was retained.

Enzyme replacement therapy reduces the presence and severity of oral manifestations by improving tooth eruption and development, decreasing premature loss of teeth, and reducing mobility and pocket depth instability [44]. In addition, McKee et al. (2011) reported that injection of human tissue-nonspecific alkaline phosphatase isozyme into HPP model mice leads to the formation of acellular cementum, suggesting prevention of accelerated tooth loss [45]. Tooth loss in the present case was divided into three categories. The first category is “tooth exfoliation during root formation”, which occurred in the maxillary primary canine and the mandibular left primary incisor and is characteristic of HPP. It has been reported that the primary incisors of HPP patients fall out without inflammation, and the root remains under formation or attached to the lost tooth [6,46].

The second category is “tooth exfoliation with root resorption”, which occurred in the maxillary primary central incisor and the primary left lateral incisor. It has been reported that before tooth loss in HPP patients, the gingival tissue gradually recedes and the height of the alveolar bone decreases; this is also characteristic of HPP [16,47]. Inflammatory root resorption is a consequence and/or complication of different clinical situations, such as pulp, periodontal, and/or periradicular infectious processes, dental trauma, orthodontic forces, or excessive occlusal forces [48]. In the present case, there were neither dental caries, dental trauma, nor orthodontic treatment. Kiselnikova et al. (2020) reported that in some HPP patients receiving asfotase alfa, teeth with Grade I mobility became stable during the treatment, and no mobility was observed in those teeth during the follow-up period, although recession of the gingival margin was present [16]. Teeth might then be lost because of root resorption resulting from periodontal infection caused by gingival recession.

The third category is “tooth exfoliation with root fracture”, which occurred in the mandibular left primary canine. Although the pulp chamber is large in patients with HPP, no closure of the pulp chamber was observed over time in the present case. Additionally, although an alveolar abscess occurred, there were no carious teeth. In XLH patients, who are also characterized as having a large pulp chamber, alveolar abscesses occur in the absence of caries or trauma [22,49]. In addition, Giuca et al. (2024) pointed out the poor enamel mineralization with the presence of cracks on the surface of the teeth in XLH patients [22]. Enlarged pulp chambers or abnormalities of enamel have also been observed in HPP patients, which may be associated with root fracture or alveolar abscess formation.

In the present case, none of the teeth were lost whose root was left completely and free of inflammation. Although tooth exfoliation during root formation occurred in the maxillary primary canine and the mandibular left primary incisor, other exfoliated teeth were lost with root resorption. These characteristics are different from those of HPP, suggesting early enzyme replacement may prevent tooth loss without root resorption in HPP patients. However, because the primary anterior teeth of this HPP patient were lost much earlier than those of healthy children, early enzyme replacement therapy did not sufficiently suppress tooth loss, and its effectiveness needs to be carefully considered. Tooth development begins in the fetus early in the second trimester [50]. Therefore, the effectiveness of enzyme replacement therapy may vary depending on the type of tooth even if it is administered immediately at birth. Yoshida et al. (2023) suggest that prenatal enzyme replacement therapy may be effective for the maxillofacial region in animal experiments [51]. On the other hand, the main concern with prenatal enzyme replacement therapy is maternal safety, with side effects including erythema, pruritus, nodules, and pain due to injection site reactions [52,53,54,55]. Future basic research is required to develop effective enzyme replacement therapy protocols.

Early enzyme replacement therapy can reduce the risk of tooth loss in HPP. However, new infection control measures, such as protection of the periodontal tissue and the enlarged pulp chamber, may be important for tooth preservation. In XLH patients, early crown restoration of teeth at the highest risk of pulp infection is strongly recommended, and these restorations include resin fissure sealing and stainless steel crowns [56]. In addition, the use of mouth protectors can minimize the occurrence of traumatic injuries to the anterior teeth [57]. These treatments may also be effective for HPP patients, as observed in our manuscript, and additional surveys will lead to formulating the guidelines for oral management for HPP patients. We continue to monitor the patient’s condition and plan to consider these treatments if necessary.

Premature loss of primary teeth can be a manifestation of systemic disease [58]. The definitive diagnosis is completed by a medical doctor; however, the characteristics in the oral region vary depending on the systemic disease. Spodzieja et al. (2022) categorized the systemic diseases that result in premature tooth loss in children into three groups: periodontal ligament destruction, such as Papillon–Lefèvre syndrome; leukocyte defects, such as cyclic neutropenia and Down syndrome; or defective development of tooth cementum, such as HPP [58]. It is important for dental professionals to understand oral findings that suspect a systemic disease and consider referring the patient to a medical doctor when dental professionals detect oral findings.

This report has some limitations. First, this is a single case report because there are few HPP patients, and it has been less than 10 years since enzyme replacement therapy was approved. Studies of pediatric patients with systemic diseases are sometimes limited by small sample sizes [59,60,61]. It is important to conduct additional surveys at multiple medical institutions with increased sample sizes. Second, the patient is in the primary dentition stage, and the status of the permanent teeth is unknown. We are continuing to monitor the patient’s progress.

## 4. Conclusions

In the present case, enzyme replacement therapy was started at 6 days of age, and the exfoliation of 11 primary teeth had occurred by the age of 4 years and 7 months. Some of these teeth had inflammatory root resorption. Radiographic examination revealed the retained roots of several primary canines that were thought to have been exfoliated. Additionally, attrition caused an alveolar abscess in the primary molar region during the follow-up period. This report highlights how enzyme replacement therapy at an early stage can prevent the loss of some primary teeth. However, infection control of large pulp chambers and periodontal tissue may pose new challenges for dental professionals.

## Figures and Tables

**Table 1:** Tooth exfoliation times.

Tooth	Time
Mandibular left primary central incisor	1 year and 3 months
Mandibular right primary central incisor	1 year and 7 months
Mandibular left primary lateral incisor	2 years
Mandibular right primary lateral incisor	2 years and 4 months
Maxillary right primary canine	2 years and 7 months
Mandibular right primary canine	2 years and 8 months
Maxillary right primary central incisor	3 years
Maxillary left primary canine	3 years
Maxillary left primary central incisor	3 years and 9 months
Mandibular left primary canine	4 years and 3 months
Maxillary left primary lateral incisor	4 years and 6 months

## Data Availability

Data are contained within this article.

## References

[1] Reis F.S., Lazaretti-Castro M. (2023). Hypophosphatasia: From birth to adulthood. Arch. Endocrinol. Metab..

[2] Tournis S., Yavropoulou M.P., Polyzos S.A., Doulgeraki A. (2021). Hypophosphatasia. J. Clin. Med..

[3] Mornet E. (2018). Hypophosphatasia. Metabolism.

[4] Michigami T., Tachikawa K., Yamazaki M., Kawai M., Kubota T., Ozono K. (2020). Hypophosphatasia in Japan: ALPL Mutation Analysis in 98 Unrelated Patients. Calcif. Tissue Int..

[5] Bloch-Zupan A., Vaysse F. (2017). Hypophosphatasia: Oral cavity and dental disorders. Arch. Pediatr..

[6] Okawa R., Nakano K. (2022). Dental manifestation and management of hypophosphatasia. Jpn. Dent. Sci. Rev..

[7] van den Bos T., Handoko G., Niehof A., Ryan L.M., Coburn S.P., Whyte M.P., Beertsen W. (2005). Cementum and dentin in hypophosphatasia. J. Dent. Res..

[8] Hollis A., Arundel P., High A., Balmer R. (2013). Current concepts in hypophosphatasia: Case report and literature review. Int. J. Paediatr. Dent..

[9] Foster B.L., Nagatomo K.J., Nociti F.H., Fong H., Dunn D., Tran A.B., Wang W., Narisawa S., Millán J.L., Somerman M.J. (2012). Central role of pyrophosphate in acellular cementum formation. PLoS ONE.

[10] Gasque K.C., Foster B.L., Kuss P., Yadav M.C., Liu J., Kiffer-Moreira T., van Elsas A., Hatch N., Somerman M.J., Millán J.L. (2015). Improvement of the skeletal and dental hypophosphatasia phenotype in Alpl-/- mice by administration of soluble (non-targeted) chimeric alkaline phosphatase. Bone.

[11] Foster B.L., Sheen C.R., Hatch N.E., Liu J., Cory E., Narisawa S., Kiffer-Moreira T., Sah R.L., Whyte M.P., Somerman M.J. (2015). Periodontal Defects in the A116T Knock-in Murine Model of Odontohypophosphatasia. J. Dent. Res..

[12] Itro A., Difalco P., Urciuolo V., Diomajuta A., Corzo L. (2005). The aesthetic and functional restoration in the case of partial edentulism in young patients. Minerva Stomatol..

[13] Akitomo T., Kusaka S., Iwamoto Y., Usuda M., Kametani M., Asao Y., Nakano M., Tachikake M., Mitsuhata C., Nomura R. (2023). Five-Year Follow-Up of a Child with Non-Syndromic Oligodontia from before the Primary Dentition Stage: A Case Report. Children.

[14] Suvarna G.S., Nadiger R.K., Guttal S.S., Shetty O. (2014). Prosthetic rehabilitation of hypophosphatasia with precision attachment retained unconventional partial denture: A case report. J. Clin. Diagn. Res..

[15] Kramer K., Chavez M.B., Tran A.T., Farah F., Tan M.H., Kolli T.N., Dos Santos E.J.L., Wimer H.F., Millán J.L., Suva L.J. (2021). Dental defects in the primary dentition associated with hypophosphatasia from biallelic ALPL mutations. Bone.

[16] Kiselnikova L., Vislobokova E., Voinova V. (2020). Dental manifestations of hypophosphatasia in children and the effects of enzyme replacement therapy on dental status: A series of clinical cases. Clin. Case Rep..

[17] Harrison H.E., Harrison H.C. (1979). Rickets and osteomalacia. Disorders of Calcium and Phosphate Metabolism in Childhood and Adolescence.

[18] Lin E.L., Gottesman G.S., McAlister W.H., Bijanki V.N., Mack K.E., Griffin D.M., Mumm S., Whyte M.P. (2020). Healing of vitamin D deficiency rickets complicating hypophosphatasia suggests a role beyond circulating mineral sufficiency for vitamin D in musculoskeletal health. Bone.

[19] Mudgade D., Srivastava H.M., Qureshi S.M.R., Handa A. (2023). Rickets—A case report. J. Oral Maxillofac. Pathol..

[20] Imel E.A. (2021). Burosumab for Pediatric X-Linked Hypophosphatemia. Curr. Osteoporos. Rep..

[21] Nguyen C., Celestin E., Chambolle D., Linglart A., Biosse Duplan M., Chaussain C., Friedlander L. (2022). Oral health-related quality of life in patients with X-linked hypophosphatemia: A qualitative exploration. Endocr. Connect..

[22] Giuca M.R. (2024). Rare diseases: A challenge in paediatric dentistry. Eur. J. Paediatr. Dent..

[23] Orimo H. (2016). Pathophysiology of hypophosphatasia and the potential role of asfotase alfa. Ther. Clin. Risk Manag..

[24] Simon S., Resch H. (2020). Treatment of hypophosphatasia. Wien. Med. Wochenschr..

[25] Simon S., Resch H., Klaushofer K., Roschger P., Zwerina J., Kocijan R. (2018). Hypophosphatasia: From Diagnosis to Treatment. Curr. Rheumatol. Rep..

[26] Jaswanthi N., Sindhu R., Nimmy P., Prabu D., RajMohan M., Bharathwaj V.V., Dhamodhar D., Sathiyapriya S. (2023). Effect of Asfotase Alfa in the Treatment of Hypophosphatasia- A Systematic Review. J. Pharm. Bioallied Sci..

[27] Schroth R.J., Long C., Lee V.H.K., Alai-Towfigh H., Rockman-Greenberg C. (2021). Dental outcomes for children receiving asfotase alfa for hypophosphatasia. Bone.

[28] Usuda M., Akitomo T., Kametani M., Kusaka S., Mitsuhata C., Nomura R. (2023). Dens invaginatus of fourteen teeth in a pediatric patient. Pediatr. Dent..

[29] Frencken J.E., Sharma P., Stenhouse L., Green D., Laverty D., Dietrich T. (2017). Global epidemiology of dental caries and severe periodontitis—A comprehensive review. J. Clin. Periodontol..

[30] Usuda M., Kametani M., Hamada M., Suehiro Y., Matayoshi S., Okawa R., Naka S., Matsumoto-Nakano M., Akitomo T., Mitsuhata C. (2023). Inhibitory Effect of Adsorption of Streptococcus mutans onto Scallop-Derived Hydroxyapatite. Int. J. Mol. Sci..

[31] Yasuda J., Yasuda H., Nomura R., Matayoshi S., Inaba H., Gongora E., Iwashita N., Shirahata S., Kaji N., Akitomo T. (2024). Investigation of periodontal disease development and *Porphyromonas gulae* FimA genotype distribution in small dogs. Sci. Rep..

[32] Jamani N.A., Ardini Y.D., Harun N.A. (2018). Neonatal tooth with Riga-Fide disease affecting breastfeeding: A case report. Int. Breastfeed. J..

[33] Bueno N.P., Bergamini M.L., Elias F.M., Braz-Silva P.H., Ferraz E.P. (2020). Unusual giant complex odontoma: A case report. J. Stomatol. Oral Maxillofac. Surg..

[34] Kartha S., Vellore K.P., Challa S.K., Vallu R., Pusuluri S. (2022). Traumatogenic Occlusion in a Pediatric Dental Patient: A Case Report. Int. J. Clin. Pediatr. Dent..

[35] Akitomo T., Asao Y., Iwamoto Y., Kusaka S., Usuda M., Kametani M., Ando T., Sakamoto S., Mitsuhata C., Kajiya M. (2023). A Third Supernumerary Tooth Occurring in the Same Region: A Case Report. Dent. J..

[36] Akitomo T., Kusaka S., Usuda M., Kametani M., Kaneki A., Nishimura T., Ogawa M., Mitsuhata C., Nomura R. (2023). Fusion of a Tooth with a Supernumerary Tooth: A Case Report and Literature Review of 35 Cases. Children.

[37] Sockanathan L., Ahmad N.S., Zakaria A.S.I. (2024). Early Detection and Interceptive Orthodontic Treatment of Impacted Canine: A Case Report. Int. J. Clin. Pediatr. Dent..

[38] Akitomo T., Asao Y., Mitsuhata C., Kozai K. (2021). A new supernumerary tooth occurring in the same region during follow-up after supernumerary tooth extraction: A case report. Pediatr. Dent..

[39] Piekoszewska-Ziętek P., Olczak-Kowalczyk D., Pańczyk-Tomaszewska M., Gozdowski D. (2022). Developmental Abnormalities of Teeth in Children With Nephrotic Syndrome. Int. Dent. J..

[40] Akitomo T., Tsuge Y., Mitsuhata C., Nomura R. (2024). A Narrative Review of the Association between Dental Abnormalities and Chemotherapy. J. Clin. Med..

[41] Okawa R., Kadota T., Matayoshi S., Nakano K. (2020). Dental Manifestations Leading to the Diagnosis of Hypophosphatasia in Two Children. J. Dent. Child.

[42] Hayashi-Sakai S., Hayashi T., Sakamoto M., Sakai J., Shimomura-Kuroki J., Nishiyama H., Katsura K., Ike M., Nikkuni Y., Nakayama M. (2016). Nondestructive Microcomputed Tomography Evaluation of Mineral Density in Exfoliated Teeth with Hypophosphatasia. Case Rep. Dent..

[43] Massler M., Schour R., Poncher H.G. (1941). Developmental pattern of the child as reflected in the calcification pattern of the teeth. Am. J. Dis. Child..

[44] Smart G., Jensen E.D., Poirier B.F., Sethi S. (2023). The impact of enzyme replacement therapy on the oral health manifestations of hypophosphatasia among children: A scoping review. Eur. Arch. Paediatr. Dent..

[45] McKee M.D., Nakano Y., Masica D.L., Gray J.J., Lemire I., Heft R., Whyte M.P., Crine P., Millán J.L. (2011). Enzyme replacement therapy prevents dental defects in a model of hypophosphatasia. J. Dent. Res..

[46] Nunes M.E. (2007). Hypophosphatasia. GeneReviews^®^.

[47] Rothenbuhler A., Linglart A. (2017). Hypophosphatasia in children and adolescents: Clinical features and treatment. Arch. Pediatr..

[48] Vieira-Andrade R.G., Drumond C.L., Alves L.P., Marques L.S., Ramos-Jorge M.L. (2012). Inflammatory root resorption in primary molars: Prevalence and associated factors. Braz. Oral Res..

[49] Seow W.K. (2003). Diagnosis and management of unusual dental abscesses in children. Aust. Dent. J..

[50] Montag A.C., Chambers C.D., Jones K.L., Dassanayake P.S., Andra S.S., Petrick L.M., Arora M., Austin C. (2022). Collaborative Initiative on Fetal Alcohol Spectrum Disorders (CIFASD). Prenatal alcohol exposure can be determined from baby teeth: Proof of concept. Birth Defects Res..

[51] Yoshida K., Ishizuka S., Nakamura-Takahashi A., Hasegawa A., Umezawa A., Koshika K., Ichinohe T., Kasahara M. (2023). Prenatal asfotase alfa-mediated enzyme replacement therapy restores delayed calcification in a severe infantile form of hypophosphatasia model mice. Eur. J. Med. Genet..

[52] Kitaoka T., Tajima T., Nagasaki K., Kikuchi T., Yamamoto K., Michigami T. (2017). Safety and efficacy of treatment with asfotase alfa in patients with hypophosphatasia: Results from a Japanese clinical trial. Clin. Endocrinol..

[53] Hofmann C.E., Harmatz P., Vockley J., Högler W., Nakayama H., Bishop N. (2019). Efficacy and safety of asfotase alfa in infants and young children with hypophosphatasia: A phase 2 open-label study. J. Clin. Endocrinol. Metab..

[54] Reis F.S., Gomes D.C., Arantes H.P., Lazaretti-Castro M. (2020). A two-year follow-up of asfotase alfa replacement in a patient with hypophosphatasia: Clinical, biochemical, and radiological evaluation. Arch. Endocrinol. Metab..

[55] Hasegawa A., Nakamura-Takahashi A., Kasahara M., Saso N., Narisawa S., Millán J.L., Samura O., Sago H., Okamoto A., Umezawa A. (2021). Prenatal enzyme replacement therapy for Akp2 (-/-) mice with lethal hypophosphatasia. Regen. Ther..

[56] Sabandal M.M., Robotta P., Bürklein S., Schäfer E. (2015). Review of the dental implications of X-linked hypophosphataemic rickets (XLHR). Clin. Oral Investig..

[57] Adekoya-Sofowora C.A. (2001). Traumatized anterior teeth in children: A review of the literature. Niger. J. Med..

[58] Spodzieja K., Olczak-Kowalczyk D. (2022). Premature Loss of Deciduous Teeth as a Symptom of Systemic Disease: A Narrative Literature Review. Int. J. Environ. Res. Public Health.

[59] Proc P., Szczepańska J., Skiba A., Zubowska M., Fendler W., Młynarski W. (2016). Dental Anomalies as Late Adverse Effect among Young Children Treated for Cancer. Cancer Res. Treat..

[60] Kametani M., Akitomo T., Usuda M., Kusaka S., Asao Y., Nakano M., Iwamoto Y., Tachikake M., Ogawa M., Kaneki A. (2024). Evaluation of Periodontal Status and Oral Health Habits with Continual Dental Support for Young Patients with Hemophilia. Appl. Sci..

[61] Akitomo T., Ogawa M., Kaneki A., Nishimura T., Usuda M., Kametani M., Kusaka S., Asao Y., Iwamoto Y., Tachikake M. (2024). Dental Abnormalities in Pediatric Patients Receiving Chemotherapy. J. Clin. Med..

